# Effect of water-DNA interactions on elastic properties of DNA self-assembled monolayers

**DOI:** 10.1038/s41598-017-00605-x

**Published:** 2017-04-03

**Authors:** Carmen M. Domínguez, Daniel Ramos, Jesús I. Mendieta-Moreno, José L. G. Fierro, Jesús Mendieta, Javier Tamayo, Montserrat Calleja

**Affiliations:** 1 Bionanomechanics Lab, Instituto de Microelectrónica de Madrid, IMM-CNM (CSIC), Isaac Newton 8 (PTM), E-28760 Tres Cantos, Madrid, Spain; 2 Molecular Modelling Group, CBMSO (CSIC-UAM), ES-28049 Madrid, Spain; 3 Departamento de Física Teórica de la Materia Condensada and Condensed Matter Physics Center (IFIMAC), UAM, ES-28049 Madrid, Spain; 4 Instituto de Catálisis y Petroleoquímica, ICP (CSIC), E-28049 Cantoblanco, Madrid Spain; 5 Departamento de Biotecnología, Universidad Francisco de Vitoria, ctra. Pozuelo-Majadahonda, km 1,800, 28223 Pozuelo de Alarcón (Madrid), Spain

## Abstract

DNA-water interactions have revealed as very important actor in DNA mechanics, from the molecular to the macroscopic scale. Given the particularly useful properties of DNA molecules to engineer novel materials through self-assembly and by bridging organic and inorganic materials, the interest in understanding DNA elasticity has crossed the boundaries of life science to reach also materials science and engineering. Here we show that thin films of DNA constructed through the self-assembly of sulfur tethered ssDNA strands demonstrate a Young’s modulus tuning range of about 10 GPa by simply varying the environment relative humidity from 0% up to 70%. We observe that the highest tuning range occurs for ssDNA grafting densities of about 3.5 × 10^13^ *molecules*/*cm* ^2^, where the distance between the molecules maximizes the water mediated interactions between the strands. Upon hybridization with the complementary strand, the DNA self-assembled monolayers significantly soften by one order of magnitude and their Young’s modulus dependency on the hydration state drastically decreases. The experimental observations are in agreement with molecular dynamics simulations.

## Introduction

DNA-based materials have been revealed as promising candidates for diverse applications such as diagnosis^1, 2^, building nanophotonic structures^3^, and protein production^4^. As a particular case, self-assembled monolayers (SAMs) of DNA biomolecules on inorganic surfaces have been employed in different technologies as microarrays^5, 6^, electrochemical^7^ and nanomechanical sensing^8, 9^. The interest of the DNA molecules both in biology and in the more recent DNA nanotechnology field has driven its study from diverse perspectives, from single molecule simulations to the experimental characterization of DNA crystals or bundles^10^. SAMs of DNA biomolecules are particularly interesting due to the capability to build monolayers at controlled grafting densities. For all the cited applications and from fundamental studies to the design of novel routes for DNA-based nanomechanical sensors or even biocompatible materials, solvation is of particular relevance. Water molecules around DNA have a two-fold role; the first hydration shell is considered integral part of the DNA structure and beyond that, water molecules are considered to act as a solvent for the polyelectrolyte^11^. The later implies changes in the DNA biomolecule persistence length that affect the mechanical properties of the DNA strand. Although single molecule mechanical properties have been experimentally addressed through optical tweezers and force spectroscopy^12–14^; still, the mechanics of 2D SAMs of DNA are yet to be explored^15, 16^. We show here that nanomechanical sensors are optimally suited to characterize averaged material mechanical properties on such 2D biomaterials.

The Young’s modulus of a material reflects the averaged effect of the stiffness of its molecular and atomic bonds. Being the DNA strands tethered to a surface, we will focus on the water junctions, which drive the intermolecular interactions in the layer. The structural water around biomolecules is key for important biochemical processes, as water molecules effectively maintain the structure and function^8, 17^ of the DNA strands. The elasticity of such layers necessarily relates to these intermolecular bonds as it also occurs in metallic glasses^18^, where the proportion of structural atoms to solvent atoms and their interactions throughout the whole material gives rise to distinctive mechanical properties^19^. It has been demonstrated^20^ that in these systems the elastic modulus is primarily determined not by the forces between the structural atoms, but by the interaction with the “solvent”, i.e. the water molecules, if we extend the analogy to DNA SAMs. It is well known that steric effects also act in the polyelectrolyte layer, as proven by the fact that the molecular surface density affects the persistence length of the strand, which also depends on the particular hydration status^12^.

**Figure 1:**
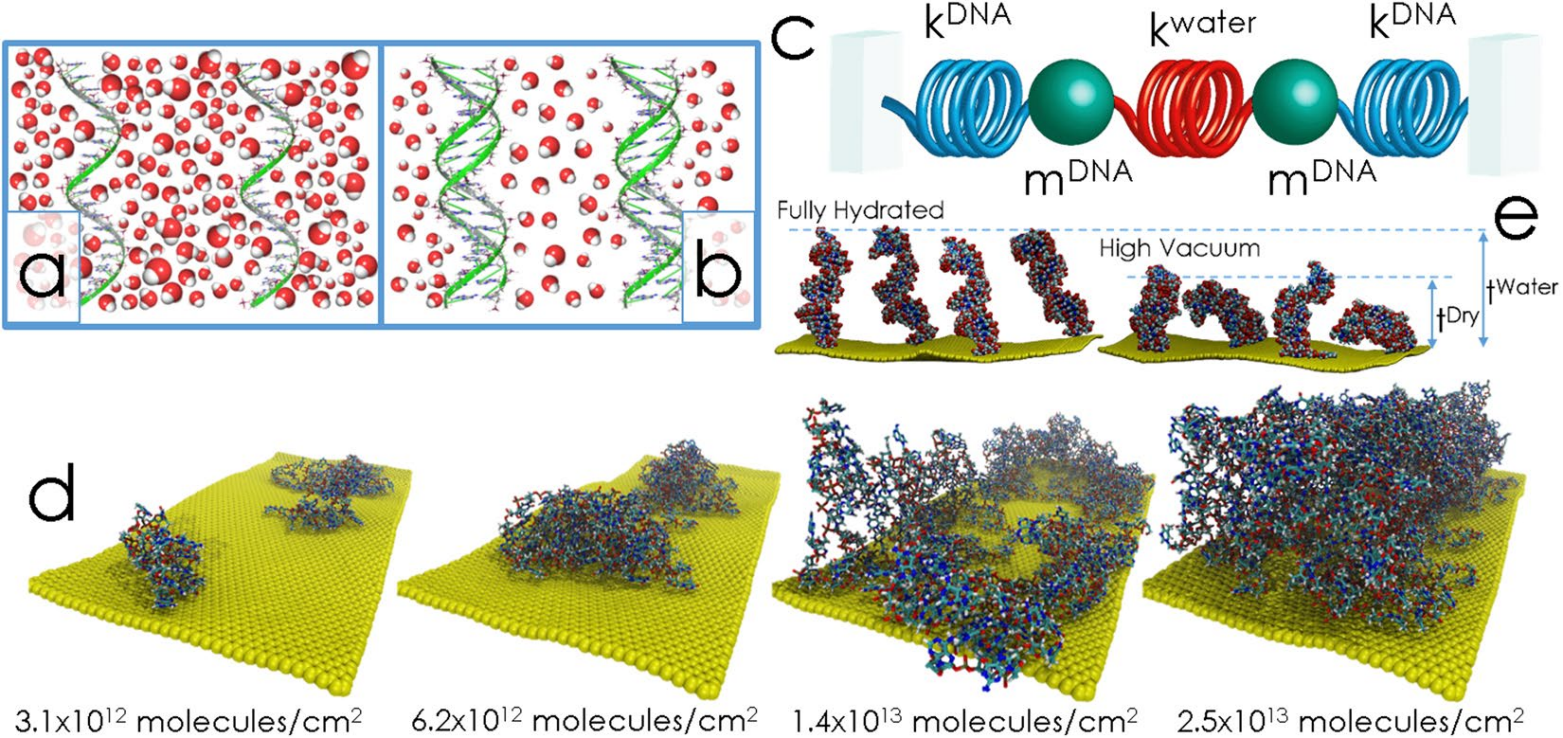
Schematics of the DNA-water interactions and mechanical analogy representation. Schematic cartoon of the hydrated ssDNA (**a**) and dsDNA (**b**). The number of water molecules depicted here is an estimation based on molecular dynamics simulations of hydrated layers. (**c**) Schematic cartoon of the mechanical analog of the DNA layer. Both the intermolecular interactions and the water molecules hold together the molecular components of the layer, giving as a result an effective spring constant. (**d**) Molecular dynamics simulations series for increasing molecular surface density from 3.1 × 10^12^ molecules/cm^2^ up to 2.5 × 10^13^ molecules/cm^2^ for fully hydrated randomly attached ssDNA molecules on a non-interacting surface of 136 nm^2^; Na^+^ ions were introduced to neutralize the excess of charge. (**e**) MD simulation showing the thickness difference between a fully hydrated DNA layer and the same layer at dry, high vacuum conditions. Note the structural effect of water molecules in the individual ssDNA molecules, see Supplementary Information for further details.

Thus, given the importance of hydration condition in this system, in the present work we simulate and experimentally study the average effect of all intermolecular forces acting in the DNA layer when changing the relative humidity of the surrounding medium. We have also varied the molecular surface grafting density of the layer with the goal of modulating intermolecular forces and the amount of water that can fit between the grafted strands due to steric effect (see schematics in Fig. 1a and b). We may depict the DNA biomolecule (ssDNA or dsDNA) as a stiff spring representing all the interaction forces, except those mediated by water molecules (i.e. Watson-Crick hydrogen bonding, base-phosphorous hydrogen bonding, van der Waals base-base interaction and base stacking), which is connected in series to the neighbor DNA molecule by a much weaker spring representing the water-mediated bonds (see schematics in Fig. 1c). Both the interactions between DNA strands and with the water molecules hold together the molecular components of the polymer-like layer, giving as a result an effective spring constant. We observe here that variations of surface density and relative humidity (RH) serve to tune the layer elasticity.

In order to account for the intermolecular forces present in the DNA SAM, we have performed molecular dynamics simulations^21–23^ (see Supplementary Information for a detailed review of the interactions considered: Watson-Crick hydrogen bonding, base-phosphorous hydrogen bonding, Van der Waals base-base interaction and base stacking). Figure 1d shows simulations of increasing numbers of randomly attached fully hydrated ssDNA molecules. As expected and also obtained from these results, the sustained angle between the strands and the substrate increases, i.e. the ssDNA molecules rise up from the substrate, as the molecular surface density increases^24^ from 3.1 × 10^12^ molecules/cm^2^ to 2.5 × 10^13^ molecules/cm^2^. The effective thickness consequently growths from 0.3 nm (corresponding to the base diameter) at low concentration, when the strands lay down on the surface, up to 1.5 nm at the maximum simulated concentration of 2.5 × 10^13^ molecules/cm^2^. These simulations were experimentally validated by AFM and XPS measurements (see Supplementary Information for additional experimental measurements and simulations, and a detailed discussion about the thickness uncertainty determination).

MD simulations at constant grafting density show the effect of the hydration on the layer thickness. By comparing the dry, high vacuum conditions with the fully hydrated layers, we note that the presence of solvent water molecules around the DNA stretch the molecule increasing the layer thickness, as can be seen from molecular dynamics simulations in Fig. 1e.

## Results and Discussion

Nanomechanical devices have demonstrated high sensitivity to changes in both the inertial mass and the stiffness^25, 26^ and they have shown an unprecedented sensitivity as mass sensors reaching impressive milestones as yoctogram resolution^27, 28^ or as force detectors with capability to detect a single spin^29^. However, their use to characterize the elasticity of thin films has been less explored^30, 31^. We are applying them here to study the above-mentioned DNA SAMs. As a model system, we have chosen ssDNA, oligo sequence 5′-CAATGCAGATACACTTTTTT-C_3_H_6_-SH-3′, grafted at different immobilization times on gold-coated silicon microcantilevers, as well as those after incubation with the complementary DNA sequence. The cantilever dimensions are 500 μm in length, 100 μm in width, and 1 μm in thickness. The resonance frequency is of 5 kHz with a mechanical quality factor of about 20 and a frequency fluctuation noise measured by the PLL of barely 50 mHz (see Supplementary Information for further details and measurements). The versatility of nanomechanical sensors^32^ is based in their two possible operational modes: the static mode, devoted to measurement of the static cantilever deflection caused by a differential surface stress^33^; and the dynamic mode, that tracks changes in resonance frequency and mechanical quality factor originated by a variation in the inertial mass or in the resonator stiffness^31^. We have developed instrumentation able to follow both microcantilever static bending and resonance frequency simultaneously that can effectively uncouple both effects (see Supplementary Information) avoiding measurement artifacts coming from nonlinearities in the instrumental response.

**Figure 2:**
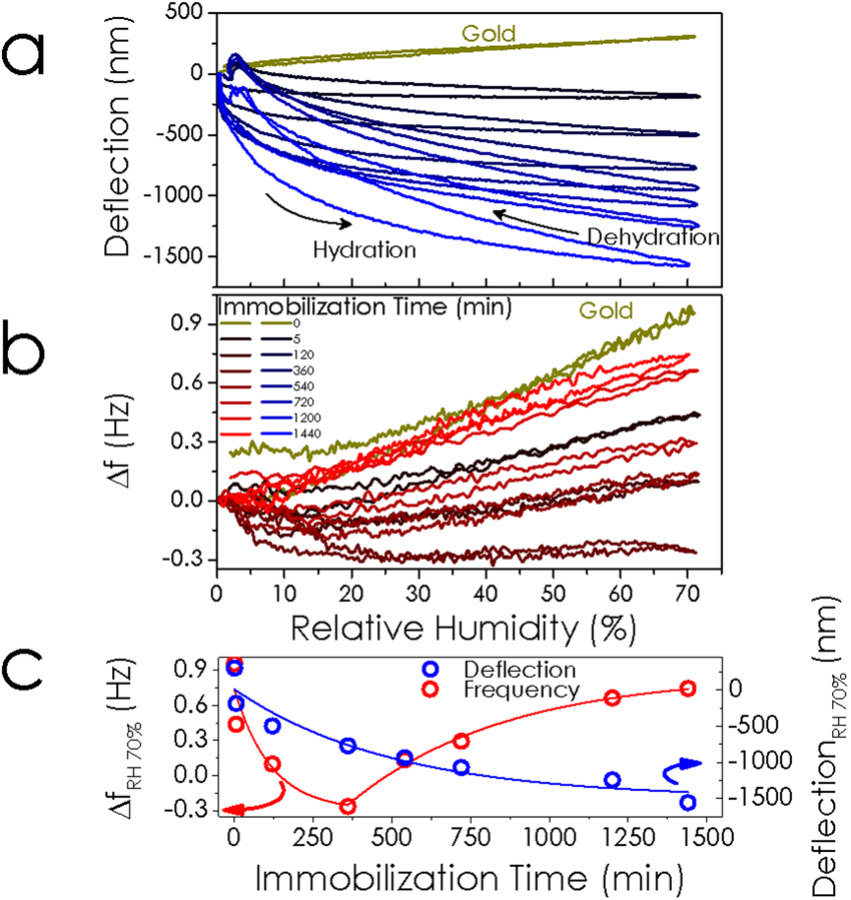
Hydration dependence of the static deflection and the fundamental resonance frequency of cantilevers for increasing packing density of the ssDNA SAM. (**a**) Cantilever static deflection variation during a hydration and dehydration cycle for a representative gold-coated silicon cantilever sensitized with thiol-modified 20-mer ssDNA molecules. For comparison, the hydration/dehydration loop for the gold-coated cantilever before functionalization is also shown (dark yellow line). The static deflection variations are measured with respect to the rest position at a relative humidity of 0%. (**b**) Cantilever fundamental resonance frequency variations as a function of the relative humidity for increasing incubation times ranging from 5 min up to 24 h. For comparison, we show the hydration/dehydration loop for the gold-coated cantilever before immobilization. Measurements were acquired simultaneously to those in (**a**) for each cantilever at every given incubation time. The resonance was measured by a homemade detection system, see Supplementary Information. (**c**) Values of the deflection (blue symbols) and the frequency shift (red symbols) at RH 70% as a function of the immobilization time.

The mechanical response of the microcantilevers grafted with ssDNA was followed upon relative humidity variations ranging from a pure dry nitrogen atmosphere up to 70% RH. Figure 2a shows the measurement of the deflection (color ranging from black to blue) by changing the relative humidity of the environmental chamber at a rate of 10.00 ± 0.08% min^−1^ while keeping constant the surrounding temperature at 298.15 ± 0.02 K. Different microcantilevers functionalized with ssDNA molecules on one surface at increasing immobilization times (none, 5 min, 120 min, 360 min, 540 min, 720 min, 1200 min, and 1440 min) are shown.

The ssDNA-immobilized cantilevers show the characteristic deflection dependence on relative humidity, which is also accompanied by hysteresis in the hydration/dehydration loop^34^. The behavior is monotonous with the immobilization time: the larger the immobilization time, the larger the cantilever deflection, ranging from −180 nm for 5 min immobilization to −1550 nm for 24 h immobilization. The dark yellow curve shows the deflection of a bare gold-coated silicon cantilever for comparison.

We have simultaneously acquired the frequency shift (red curves from dark to light in Fig. 2b) for the same cantilevers shown in Fig. 2a. In order to easily follow these changes, we depict in Fig. 2b the resonance frequency variation Δf(RH) = f(RH) − f(0%). When compared to the deflection curves, there are two distinctive features for the frequency measurements: the hysteresis is almost negligible and there is a non-monotonic behavior of the frequency shift as a function of the immobilization time. Figure 2c summarizes the values of the deflection (blue symbols) and the frequency shift (red symbols) at RH 70%, highlighting this difference. The line is just a guide for the eye.

For low immobilization times, and thus, low grafting density, the resonance frequency shift is lower than for the bare gold system (dark yellow curve), reaching a minimum at 360 minutes of incubation time. This difference increases again with the immobilization time from that point onwards, red symbols in Fig. 2c. This non-monotonous dependency of the resonance frequency with immobilization time (a decrease followed by an increase) strongly suggests the existence of a competition between two opposite mechanisms acting at the same time. As we know, the resonance frequency could be understood as the result from an energy balance between the kinetic and the potential energy. While the kinetic energy is only affected by the mass of the resonator, the potential energy is related to the stiffness of the resonator^30^.

**Figure 3:**
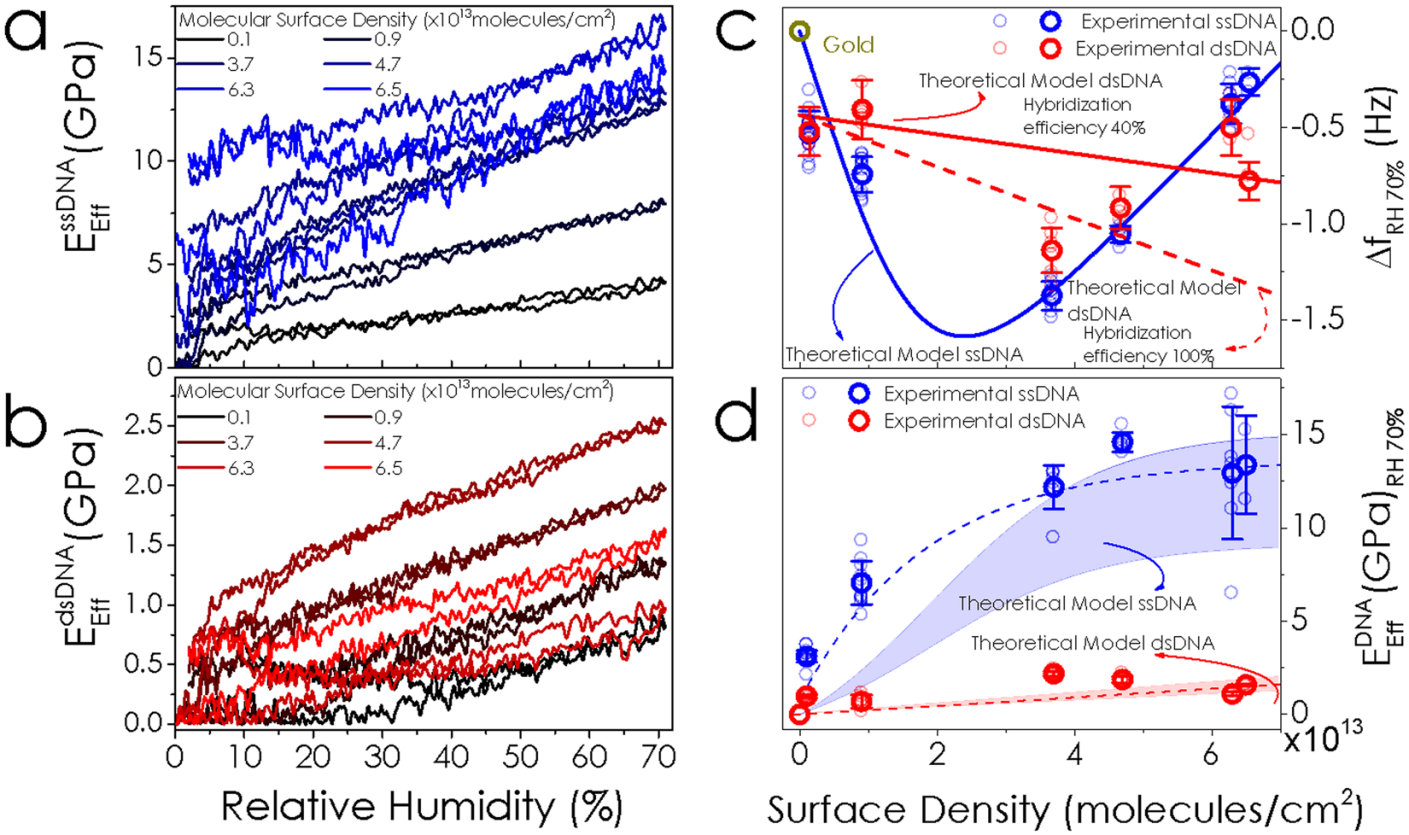
Modulation of the effective Young’s modulus of the DNA layer. Experimental tracking of the ssDNA (**a**) and dsDNA (**b**) Young’s modulus during a hydration and dehydration cycle for a cantilever immobilized with increasing surface density of DNA molecules. By increasing the environmental relative humidity, the effective Young’s modulus linearly increases, showing no-hysteresis features. The effective Young’s modulus for the dsDNA is not only one order of magnitude lower but also less sensitive to external humidity variation due to the double helix shielding effect. (**c**) Theoretical model (lines) and experimental measurement (symbols) of frequency shifts for fully hydrated DNA. The frequency shift for ssDNA (blue circles) decreases for low surface coverage until a critical value, where it increases again. The experimental shifts in frequency for varying dsDNA coverage are represented in red circles. The discrepancy with the theoretically calculated shift (red dashed line) could be attributed to the experimentally inhomogeneous hybridization surface coverage. The red solid line considers that the hybridization efficiency is of about 40% whereas there is a nonspecific adsorption of about 15% of DNA strands. (**d**) Young’s modulus for fully hydrated ssDNA (blue symbols) and dsDNA (red symbols) as a function of molecular surface density. The theoretical calculations (bluish and reddish areas respectively for ssDNA and dsDNA) match the experimental values (open symbols). While the Young’s modulus for the ssDNA reaches an asymptotic value of ~15 GPa, the value for the dsDNA only reaches a maximum value of ~2 GPa due the double helix effect.

Figure 3a shows the Young’s modulus variation for increasing surface densities of the ssDNA layers during a hydration and dehydration cycle. The molecular surface densities (calculated from XPS measurements, see Supplementary Information for further details and measurements) range from 0.1 × 10^13^ *molecules*/*cm* ^2^ up to 6.5 × 10^13^ *molecules*/*cm* ^2^, shown as blue lines from dark blue to light blue. The effective Young’s modulus, ${E}_{eff}^{DNA}$, has been calculated from the experimentally measured frequency shift. These experimental calculations^35, 36^ take into account the mechanical properties of the cantilever: mass density, geometrical dimensions and Young’s modulus of the silicon; as well as the DNA mass density, the layer thickness and the number of water molecules per DNA strand for each molecular surface density predicted by MD simulations. See Supplementary Information for a detailed discussion about the determination of these parameters. Assuming that the adsorbate thickness (~1 nm) is three orders of magnitude smaller than the cantilever thickness (~1 μm), by expanding the resonance in power series of the thickness ratio^35^, we obtain at second order the following expression

1 $$\begin{array}{rcl}{\rm{\Delta }}\omega /{\omega }_{0} & \cong  & 1/2(3{E}_{eff}^{DNA}/{E}_{c}-{\rho }^{DNA}/{\rho }_{c})({t}^{DNA}/{t}_{c})+3/8[{({\rho }^{DNA}/{\rho }_{c})}^{2}\\  &  & +2{E}_{eff}^{DNA}/{E}_{c}(4-{\rho }^{DNA}/{\rho }_{c})-7{({E}_{eff}^{DNA}/{E}_{c})}^{2}]{({t}^{DNA}/{t}_{c})}^{2}\end{array}$$

where the subscript *c* refers to the cantilever, being *E*_*c*_ the silicon Young’s modulus, 169 GPa, *ρ*_*c*_ the silicon mass density, 2330 *kg* × *m* ^−3^, and *t*_*c*_ the thickness, 1 *μm*. The effective Young’s modulus of the DNA monolayer, ${E}_{eff}^{DNA}$, depicted in Fig. 3a and b has been obtained by simply solving for it in this approximate expression. As it can be seen from the figure, ${E}_{eff}^{DNA}$ linearly increases with the environmental relative humidity, showing no-hysteresis features during the hydration/dehydration cycle, indicating its elasticity fine tuning capability. Note that the Young’s modulus is zero for low molecular surface density at the dry estate; whereas as the surface density increases, the dry point is close to 10 GPa for ssDNA. This is due to the intermolecular interactions rising between the DNA strands, which strongly depend on the molecular separation, being negligible at low surface density where the distance between the molecules is sufficiently large. Particularly remarkable is the case of the experimental measurement taken at 3.7 × 10^13^ *molecules*/*cm* ^2^, showing a Young’s modulus tuning range of about 10 GPa, from ~5 GPa up to ~15 GPa for RH variations between 0% and 70%. Taking into account the DNA layer thickness uncertainty as main error source (see Supplementary Information for further details), the error in the Young’s modulus determination can be up to 33%, that is ${E}_{eff}^{DNA}(3.7\times {10}^{13}molecules/c{{m}^{2}}_{RH=70 \% })=12.3\pm 4.1\,GPa$. This grafting density maximizes the water-mediated interactions between the strands, while for increased surface densities, steric effects become more relevant. Figure 3b shows the Young’s modulus for increasing surface densities of the same ssDNA modified microcantilever shown in Fig. 3a but after hybridization with the complementary sequence. The elastic behavior of the dsDNA layers during hydration and dehydration cycles is very different. The double helix shielding effect implies attenuation of the water mediated interactions, which is translated into one order of magnitude lower Young’s modulus than for the corresponding ssDNA surface density; besides this, note that the dry point Young’s modulus is below 2 GPa for all the surface densities measured.

Figure 3c shows the experimentally measured resonance frequency shifts as a function of the molecular surface density referenced to the bare gold coated cantilever resonance frequency of both the ssDNA (blue symbols) and the subsequently hybridized monolayer (red symbols). For low molecular packing the interaction between the ssDNA strands is almost negligible, therefore, the effective Young’s moduli at these surface densities are too small to induce a measurable frequency shift. Thus, the fundamental resonance shifts to lower frequencies only due to the added mass of water molecules. However, as long as the molecular surface density increases, the intermolecular forces raise (both the thickness and the effective Young’s modulus of the layer increases) stiffening the cantilever-DNA system and consequently shifting the resonance to higher frequencies. The critical molecular surface coverage where the stiffness cancels the negative shifting of the added mass takes place at 30% of maximum surface coverage (approximately at 2.5 × 10^13^ *molecules*/*cm* ^2^). The corresponding simulated frequency shift is shown in the figure as a solid line (blue for the ssDNA and red for the dsDNA). Note that whereas the frequency shift for the ssDNA shows a non-monotonic behavior, the dsDNA shows a monotonously negative shift with the grafting density. Since the Young’s modulus of the dsDNA is one order of magnitude lower than the Young’s modulus of the ssDNA, the frequency shift is mainly dominated by the added mass effect, shifting the resonance to lower frequencies. The small discrepancy between the experimental results and the theoretical frequency shift for the hybridized layer (red dashed line) could be attributed to the inherent inhomogeneity in the experimental hybridization efficiency, that it is known to decrease for increasing packing density, whereas we have theoretically assumed a fully hybridized layer (red dotted line). Therefore, we have used the above described formula of the frequency shift (equation 1) to do a fitting to the experimental values by setting free two parameters: the hybridization efficiency and the added mass. The result of the fitting is shown as a red solid line in Fig. 3c, corresponding to a hybridization efficiency of 40% and an excess of mass (attributed to unspecific adsorption) of 15%.

Figure 3d shows the effective Young’s modulus for fully hydrated ssDNA (blue circles) and dsDNA (red circles) experimentally calculated by solving the resonance frequency expression (equation 1) for the Young’s modulus for the experimental resonance shifts at 70% relative humidity for each grafting density shown in Fig. 3c. The fully hydrated DNA layer thickness and the mass density were calculated by MD (see Supplementary Information for further details). For comparison, we have also included the theoretical values for the Young’s modulus derived from bending curves that we have simulated by MD (see Supplementary Information), shown as blue and red areas in Fig. 3d. The second derivative of the energy bending curves gives the spring constant of the system, ${k}_{eff}^{DNA}$, which is used to calculate the effective Young’s modulus of the DNA layer, ${E}_{eff}^{DNA}$, by simply solving ${E}_{eff}^{DNA}=4{L}^{3}/w{t}^{3}{k}_{eff}^{DNA}$, where *L*, *w* and *t* are the length, the width and the thickness of the cantilever. Experimental fitting to the data shown in Fig. 3d gives a value of ${E}_{eff}^{ssDNA}=1.5/(1.22\times {10}^{-10}+2.29\times {10}^{-9}{e}^{-1.42\times {10}^{13}{n}_{DNA}})GPa$ for the ssDNA; and ${E}_{eff}^{dsDNA}=4.59\times {10}^{6}+1.35\times {10}^{-6}{n}_{DNA}\,GPa,$ for the dsDNA.

${E}_{eff}^{DNA}$ increases with the surface density; however, whereas in the ssDNA layer (blue solid circles) reaches an asymptotic maximum value of about 15 GPa following a Boltzmann growth curve, the effective Young’s modulus for dsDNA (red solid circles) is one order of magnitude lower, ~2 GPa. The shadowed areas correspond to the theoretical uncertainty that comes from the fitting parameters in the determination of the Young’s moduli from the MD simulated bending curves in Fig. 3c. As it can be clearly seen from the figure, the experimental values follow the theoretical trends, showing that the observed tuning of the resonance frequency and thus, ${E}_{eff}^{DNA}$, arises from the intermolecular forces predicted by MD simulations.

## Summary and Conclusions

In light of these results, we can confirm that ssDNA monolayers attribute on having reconfigurable elasticity with a Young’s modulus tuning range of about 10 GPa that can be scanned by simply changing the surrounding relative humidity. This reconfigurable elasticity could be useful in many applications, from the design of humidity responding switches or engines^37^, to controlled delivery of enzymes or chemicals encaged on DNA SAMs that can be liberated at threshold ambient conditions or surfaces with tunable Young’s modulus for the control of cell adhesion and cell mechanosensation studies^38–40^. Besides this, the presented active layer could also be used as a DNA hybridization sensor, given the unambiguous response provided by the significant change in Young’s modulus for hybridization assays at a wide range of conditions for molecular surface density and environmental humidity.

## Methods

### Experimental setup

The read-out is based on the optical beam deflection method. A laser is focused onto the free end of the cantilever beam and its reflection is collected by a quadrant photodetector or by a position sensitive detector (PSD). It is known that the response of a PSD is not uniform along its whole surface; therefore, a static cantilever bending, which moves the laser spot at the surface of the detector, will induce a non-real shift in the measured resonance frequency. In order to prevent this undesirable measurement artifact, we have introduced in the optical path two mirrors actuated by an automatized motor in a feedback closed loop configuration: the output of the PSD is converted into a voltage input signal to the motors controlling the mirror angles in such a way that the change of the angle maintains the laser spot at the central point of the detector surface all the time. Therefore, the input signal of the mirror angle is translated into the static deflection of the cantilever, whereas the resonance frequency obtained by the Fast Fourier Transform (FFT) of the signal coming from the spot, always at the center of the PSD, is free from undesirable artifacts. The output signal of the photodetector is split up into two different signals, one is injected into the feedback loop controlling the mirrors and the other one is analyzed by a locking amplifier with a phase locked loop (PLL).

### Molecular dynamics (MD) simulation

In order to account only for the lateral intermolecular forces, not for the surface-molecule attraction, to simulate the DNA molecular absorption it is necessary to choose a non-interacting surface; therefore, a graphene sheet of 8 × 17 nm has been used as absorption substrate. Different numbers of DNA strands have been attached to the surface through a thioether bond to mimic the experimental conditions: for the ssDNA 4, 8, 18 and 32 molecules were uniformly distributed on the surface; and 4, 8 and 14 molecules were attached for the dsDNA. MD simulations were performed using PMEMD module of AMBER11 package. The system was solvated using a box of TIP3 waters. In each system, a minimization of 10000 steps was performed followed by a heating phase of 200 ps were the temperature was raised from 100 to 300 K. After this thermal equilibration, unrestrained MD was performed for 20 ns in each case. To simulate the bending experiments to account for the flexibility of the ssDNA and the dsDNA a potential of mean force (PMF) was performed with the WHAM method. To construct the PMF we made 55-umbrella sampling where we folded the surface in step of 0.5 Angstroms. Two force field parameters were used for all simulations: general amber force field ‘gaff’ was used for the graphene atom and parm99sb for the rest of the atoms.

### X-ray photoelectron spectra (XPS) measurements

X-ray photoelectron spectra (XPS) were recorded using a Escalab 200 R (VG, UK) electron spectrometer equipped with a hemispherical analyzer, operating in the constant pass energy mode, and a MgKa (hν = 1253.6 eV, 1 eV =  1.603×10^−19^ *J*) X-ray source operated at 10 mA and 12 kV. The detection angle of photoelectrons was 60° to the surface of the specimen. The spectrometer was calibrated against Au4f7/2 line at 84.0 eV using a gold sheet and Cu2p3/2 at 932.5 eV from a copper sheet. Charge effects on the samples were removed by taking the C1s line from adventitious carbon at 284.8 eV. In order to estimate the photoelectron peak intensities, the background was subtracted from the measured spectra according to the Shirley method and using a combination of Gaussian and Lorentzian lines (90G-10L). The relative surface atomic ratios were determined from the corresponding peak intensities, corrected with tabulated atomicsensitivity factors. The reproducibility of the results was confirmed several times under the same conditions.

### XPS thickness estimation

In order to determine the number of molecules at the cantilever surface we have performed a quantitative characterization of the DNA film by X-ray photoelectron spectroscopy (XPS). The presence of nitrogen atoms is typically used as the experimental indicator of adsorbed DNA; however, since the used buffer in the immobilization is an unspecific source of nitrogen we have chosen the phosphorous as signature indicator. The signal coming from the gold 4 f peak is attenuated as the immobilization time for the ssDNA is increased. From this attenuation, it is possible to calculate the actual thickness of the DNA layer by using the clean Au4f spectrum as reference. Then, the calculated thicknesses are used to correct the measured XPS peak ratios of the N and P atoms for attenuation. In order to do this, we have to calculate the practical effective attenuation length (PEAL, L_Au_) for electrons in the film using a reference film, whose thickness we have measured by atomic force microscopy. The relationship between the intensity of the XPS peak, *I*_*Au*_, and the thickness, *t*, is given by ${I}_{Au}={{I}_{Au}}^{0}exp(-t/{L}_{Au})$.

### Buffers and Solutions

All buffers were prepared using molecular biology grade water. The immobilization buffer consisted of 1 × Tris- EDTA (which consists of 10 mM Tris-HCl and 1 mM disodium EDTA) with NaCl 1 M and pH 7.5. In order to remove the dissolved oxygen, and thus minimize thiol oxidation, the buffer was degassed by simultaneous sonication and bubble extraction with a vacuum pump for 20 min. Thiolated DNA aliquots were prepared with the degassed 1 × TE-NaCl 1 M buffer. Low and high stringency wash buffers contained 0.1% SDS and 2 × SSC and 0.5 × SSC, respectively.

### Surface Functionalization

Prior to use, cantilever arrays were immersed in acetone and isopropyl alcohol and subsequently dried under a stream of dry nitrogen, and irradiated in a UV-ozone cleaner for 1 h. Then, the corresponding thiolated DNA was diluted in the immobilization buffer to a final concentration of 5 μM. The cantilever arrays were incubated in each DNA solution at different times (5, 120, 360, 540, 720, 1200, and 1440 min) at 25 °C. Afterward, the arrays were cleaned with low and high stringency hybridization wash buffers to wash out the physisorbed DNA away from the microcantilever surface and finally rinsed with plenty of Milli-Q water. Cleansing steps were carried out at 25 °C as well.

### Hybridization Conditions

The hybridization was performed at 25 °C overnight with the complementary sequence at a final concentration of 1 μM. The hybridization conditions were set to optimize the hybridization efficiency. After hybridization, cantilever arrays were cleaned with low and high stringency hybridization wash buffers and extensively rinsed with plenty of Milli-Q water.

### Calculation of the Young’s modulus of the DNA layer

From the theoretically simulated bending experiments, the effective spring constant of the graphene-DNA system, ${k}_{eff}^{g-DNA}$, is calculated as the second derivative of the energy curves; which will be translated into an effective Young’s modulus value, ${E}_{eff}^{g-DNA}$ by taking into consideration the actual dimensions of the cantilever device used in the experiments, ${E}_{eff}^{graph-DNA}=4{L}^{3}/w{t}^{3}{k}_{eff}^{graph-DNA}$. Finally, the Young’s modulus of the DNA will be decoupled from the system graphene-DNA by solving the following relation:

$${E}_{eff}^{g-DNA}=\frac{{E}_{g}^{2}{t}_{g}^{4}+{E}_{DNA}^{2}{t}_{DNA}^{4}(x)+2{E}_{g}{E}_{DNA}{t}_{g}{t}_{DNA}(x)[2{t}_{g}^{2}+2{t}_{DNA}^{2}(x)+3{t}_{g}{t}_{DNA}(x)]}{{E}_{g}{t}_{g}+{E}_{DNA}{t}_{DNA}(x)}$$

where the subscripts *g* and *DNA* refer respectively to the graphene and DNA. Note that both the thickness, *t*, and the Young’s modulus, *E*, of the DNA layer depend on the molecular surface density, *x*. This dependency arises from the MD bending data and it has also been experimentally observed in this work.

## Electronic supplementary material

Supplementary Info File #1
