# Supplementary material for: Effect of water-DNA interactions on elastic properties of DNA self-assembled monolayers
Source: Sci Rep. 2017 Apr 3;7:536. doi: 10.1038/s41598-017-00605-x (PMC5428875; doi:10.1038/s41598-017-00605-x)
Supplement: Supplementary file 1 — Supplementary Info File #1 [file 41598_2017_605_MOESM1_ESM.pdf]

SUPPLEMENTARY INFORMATION

Effect of water-DNA interactions on elastic properties of DNA self-assembled monolayers

Carmen M. Domínguez[1*], Daniel Ramos[1*a], Jesús I. Mendieta-Moreno[2,3], José L. G. Fierro[4], Jesús Mendieta[2,5], Javier Tamayo[1] and Montserrat Calleja[1]

[1]Bionanomechanics Lab, Instituto de Microelctrónica de Madrid, IMM-CNM (CSIC), Isaac Newton 8 (PTM), E-28760 Tres Cantos, Madrid, Spain. [2]Molecular Modelling Group, CBMSO (CSIC-UAM), ES-28049 Madrid, Spain. [3]Departamento de Física Teórica de la Materia Condensada and Condensed Matter Physics Center (IFIMAC), UAM, ES-28049 Madrid, Spain. [4]Instituto de Catálisis y Petroleoquímica, ICP (CSIC), E-28049 Cantoblanco, Madrid, Spain. [5]Departamento de Biotecnología, Universidad Francisco de Vitoria, ctra. Pozuelo – Majadahonda, km 1,800, 28223 Pozuelo de Alarcón (Madrid), Spain.

*These authors contributed equally to this work. [a] e-mail: daniel.ramos@csic.es

S1. Cantilever Platform

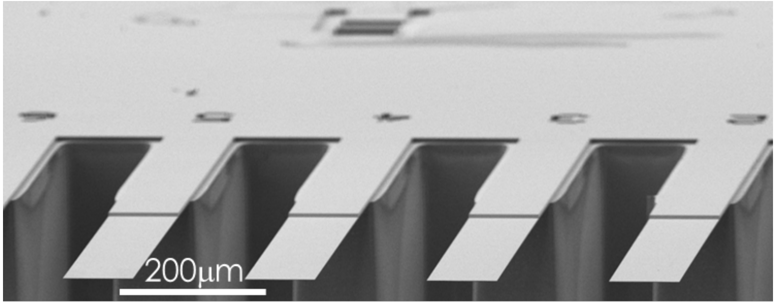

Figure S1 | Scanning Electron Microscope image of the cantilever chip used in the experiments.

Arrays of eight silicon cantilevers with 20 nm of gold coating and a Cr adhesion layer used in this work were purchased from Concentris GmbH (Basel, Swizertland). Their dimensions are 500 µm long, 100 µm wide and 1 µm thick.

S2. Experimental setup

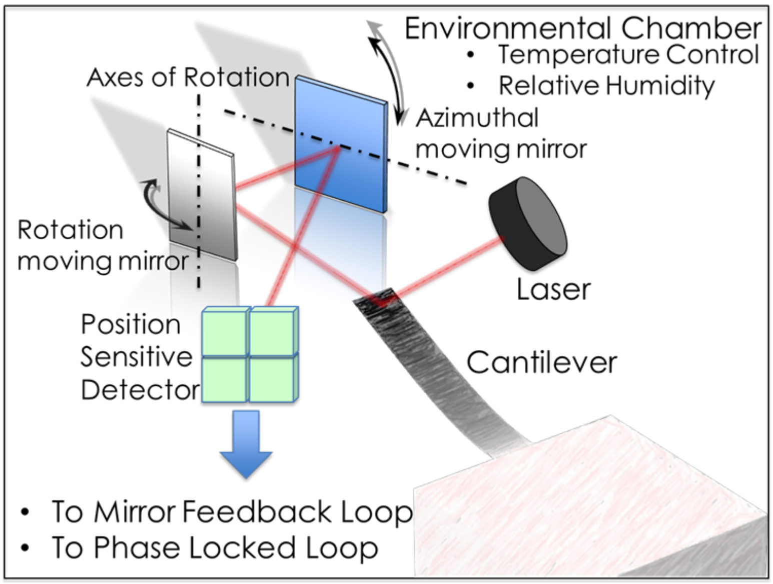

Figure S2 | Schematics of the experimental setup. Homemade experimental set-up for nanomechanical resonator measurement. The reflected laser spot from the cantilever tip is collected by a position sensitive detector (PSD) instead the usual segmented photodiode. In order to avoid the nonlinear response of the PSD the output voltage is used to actuate on angular galvo mirrors in a closed-loop configuration controlling two different angles of the optical path to keep the laser spot on the center position of the PSD sensor surface. The static deflection of the cantilever is obtained by means of the mirror input voltage, whereas a phase locked loop unit measures the resonance frequency.

In the optical beam deflection method, a laser is focused onto the free end of a cantilever beam and its reflection is collected by a quadrant photodetector or by a position sensitive detector (PSD). However, it is known that the response of a PSD is not uniform along its whole surface; therefore, a static cantilever bending, which moves the laser spot at the surface of the detector, will induce a non-real shift in the measured resonance frequency. In order to prevent this undesirable measurement artifact, we have introduced two mirrors in the optical path actuated by an automatized motor in a feedback closed loop configuration: the output of the PSD is converted into a voltage input signal to the motors controlling the mirror angles in such a way that the change of the angle maintains the laser spot at the central point of the detector surface all the time, Fig. S2. Therefore, the input signal of the mirror angle is translated into the static deflection of the cantilever, whereas the resonance frequency obtained by the Fast Fourier Transform (FFT) of the signal coming from the spot at the center of the PSD is free from undesirable artifacts. The output signal of the photodetector is split up into two different signals, one is injected into the

feedback loop controlling the mirrors and the other one is analyzed by a locking amplifier with a phase locked loop (PLL).

S3. Dynamic Characterization

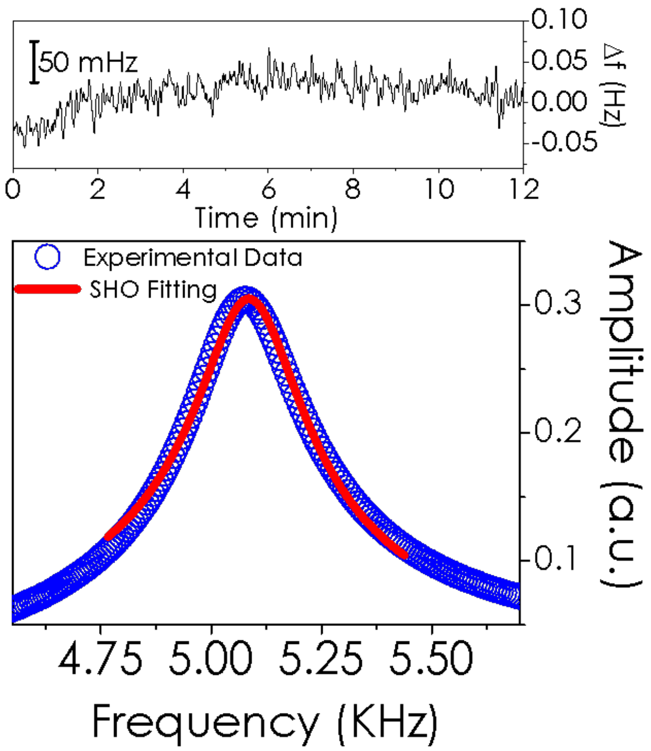

Figure S3 | Dynamic characterization of a typical cantilever used in the experiments. The upper chart shows a real-time measurement of the fundamental resonance frequency using the PLL scheme described above indicating a noise of barely 50 mHz, and the spectrum of the fundamental mechanical resonance of a typical cantilever used in the experiments showing a frequency of 5 kHz and a mechanical quality factor of 20.

In order to demonstrate the low noise level, and reproducibility of the dynamic characterization, the upper chart in Fig. S3 shows a sample of the frequency measurement obtained by the PLL, with a frequency noise of barely 50 mHz. By using the locking amplifier, the signal-to-noise ratio of the resonance peak is enhanced reaching a value of 6 at resonance frequency of 5.1 kHz with a mechanical quality factor of about 20, shown in the lower chart of Fig. S3.

S4. X-ray photoelectron spectra (XPS) measurements

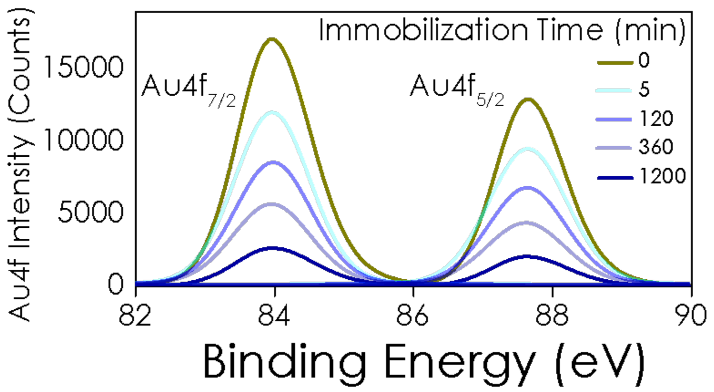

Figure S4 | XPS measurements of the gold intensity. Intensity of the Au4f peaks for increasing immobilization time of the ssDNA ranging from 5 min up to 1200 min. As long as the immobilization time increases, the packaging of the DNA layer increases attenuating the gold signal.

X-ray photoelectron spectra (XPS) were recorded using a Escalab 200R (VG, UK) electron spectrometer equipped with a hemispherical analyzer, operating in the constant pass energy mode, and a MgKα (hν = 1253.6 eV, 1 eV = $1.603\times10^{-19}\,J$) X-ray source operated at 10 mA and 12 kV. The detection angle of photoelectrons was 60° to the surface of the specimen. The spectrometer was calibrated against $Au4f_{7/2}$ line at 84.0 eV using a gold sheet and $Cu2p_{3/2}$ at 932.5 eV from a copper sheet. Charge effects on the samples were removed by taking the C1s line from adventitious carbon at 284.8 eV. In order to estimate the photoelectron peak intensities, the background was subtracted from the measured spectra according to the Shirley method and using a combination of Gaussian and Lorentzian lines (90G-10L). The relative surface atomic ratios were determined from the corresponding peak intensities, corrected with tabulated atomic sensitivity factors. The reproducibility of the results was confirmed several times under the same conditions.

In order to determine the number of molecules at the cantilever surface we have performed a quantitative characterization of the DNA film by X-ray photoelectron spectroscopy (XPS). The presence of nitrogen atoms is typically used as the experimental indicator of adsorbed DNA; however, since the used buffers in the immobilization and subsequent cleaning process are unspecific sources of nitrogen we have chosen the phosphorous as signature indicator. The signal coming from the gold 4f peak is attenuated as the immobilization time for the ssDNA is increased. From this attenuation, it is possible to calculate the actual thickness of the DNA layer by using the clean Au4f spectrum as reference. Then, the calculated thicknesses are used to correct the measured XPS peak ratios of

the N and P atoms for attenuation. In order to do this, we have to calculate the practical effective attenuation length (PEAL, L_{Au}) for electrons in the film using a reference film, whose thickness we have measured by atomic force microscopy. The relationship between the intensity of the XPS peak, I_{Au}, and the thickness, t, is given by $I_{Au} = I_{Au}{}^{0} exp(-t/L_{Au})$.

Table S1 shows the results obtained for the surface coverage by looking at the P 2p peak intensity relative to the Au 4f peak. Although the presence of nitrogen atoms is typically used as the experimental indicator of adsorbed DNA, since the used buffers in the immobilization and subsequent cleaning process are unspecific sources of nitrogen we have used the phosphorous as signature indicator.

Incubation time (min)	DNA film thickness (nm)	Atomic density of P relative to Au (N_P/N_{AU})	Relative P coverage ($\theta P/N_{AU}$)	DNA coverage n (molecules/cm^2)
5	0,2286	0,02101	0,00480	$1,39255x10^{12}$
120	0,4260	0,07359	0,03135	$9,09113x10^{12}$
360	0,7339	0,17255	0,12664	$3,67243x10^{13}$
540	0,8955	0,17988	0,16108	$4,67133x10^{13}$
720	1,0140	0,18045	0,18298	$5,30640x10^{13}$
1200	1,2000	0,18050	0,21660	$6,28140x10^{13}$
1440	1,2503	0,18050	0,22568	$6,54469x10^{13}$

Table S1 | Molecular surface density determination. By using the number of P atoms per Au atoms N_P/N_{Au} and the relative coverage $\theta_P = N_P t_{DNA}$, the surface density of molecules is given by [Petrovykh, D. Y., Kimura-Suda, H., Tarlov, M. J. & Whitman, L. J. Quantitative Characterization of DNA Films by X-ray Photoelectron Spectroscopy. *Langmuir* **20**, 429-440, doi:10.1021/la034944o (2004)] $n_{DNA} = \theta_P/N_{Au} \times 11.78 \times 10^{13}$ molecules/cm^2

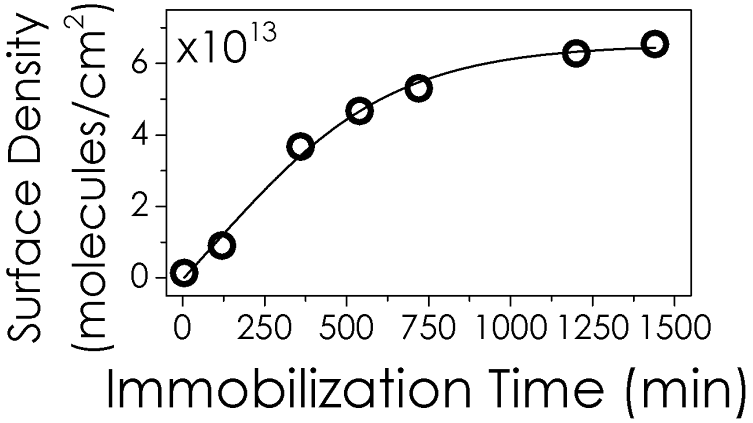

Figure S5 | XPS measurements of the molecular surface density. Molecular surface density estimation by XPS measurements as a function of the immobilization time. Note the saturation at $6.5x10^{13}$ molecules/cm^2.

Fig. S6 shows the thickness of DNA layers calculated from XPS measurements as blue and red circles for ssDNA and dsDNA, respectively. As it was previously explained, thickness was obtained by means of the Au 4f peak attenuation (the gold signal exponentially depends on the inverse of the thickness). The larger the immobilization time, the thicker the ssDNA layer. This is due to the molecular interactions between the strands that arise when the molecular surface density grows[1]. The different intermolecular forces between the strands (base stacking and water-mediated bonding through hydrogen bonds between bases and base-phosphorous) act stabilizing the structure and stretching the DNA molecules, which at low concentration are coiled and laying down on the surface, due to the attraction between the bases and gold atoms[2] corresponding to a thickness of about the base diameter (0.3 nm). Thickness asymptotically grows up to 1.2 nm for the highly packed monolayer. A non-linear curve fitting was used to determine the thickness in nanometers as a function of the immobilization time in minutes, giving as a result: $t_{ssDNA} = 1.27 + (-725.54 - 1.27)/\left(1 + -e^{(t+2853.58)/442.43}\right)\, nm$; where t is the time in minutes. The simulated thickness of the ssDNA (bluish area) shows a good agreement with the experimental measurements, see below for further details. The experimental measurements of the thickness of the dsDNA as a function of the immobilization time in minutes follows a linear trend, $t_{dsDNA} = 0.98 + 1.51\times10^{-4} t\, nm$, within the experimental window.

The light red area in Fig. S6 represents the simulated thickness of dsDNA showing a significantly larger value than for the ssDNA, of about 3 nm, and an almost negligible dependence with immobilization time, at very low concentration there is a huge contribution of the naked surface, where there are no DNA molecules, contributing to lower the mean value of the thickness. This can be explained as the dsDNA strands have a much larger persistence length and thus are known to have a smaller dependence on crowding effects[3]. We interpret the discrepancy between the experimental values and the predictions of the MD simulations as an effect of the well documented decrease in hybridization efficiency for high grafting densities, while we have considered for the MD simulations a hybridization efficiency of 100%.

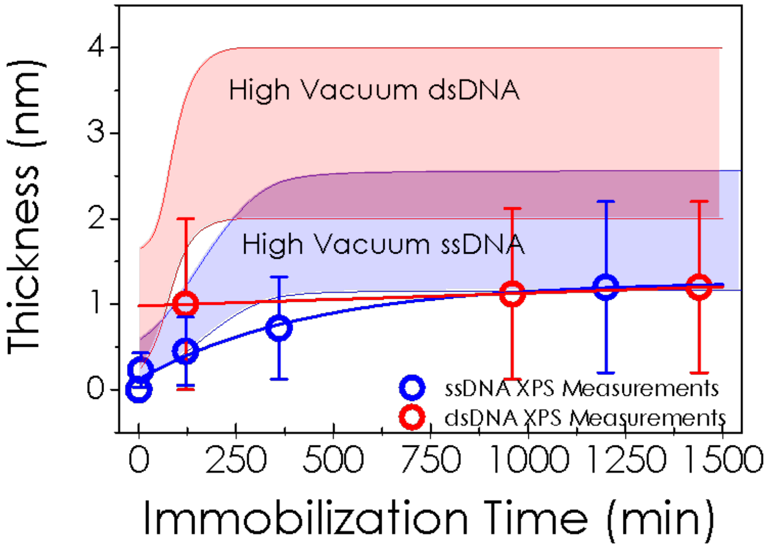

Figure S6 | Comparison between Molecular dynamics simulations of the DNA layer thickness and the XPS measurements.

S5. Molecular Dynamics Simulations. Hydration Shell

In order to account only for the lateral intermolecular forces, not for the surface-molecule attraction, to simulate the DNA molecular absorption we have chosen a non-interacting surface; therefore, a graphene sheet of 8x17 nm has been used as a modelling absorption substrate. Different numbers of DNA strands have been attached to the surface through a thioether bond to mimic the experimental conditions: for the ssDNA 4, 8, 18 and 32 molecules were uniformly distributed on the surface; and 4, 8 and 14 molecules were attached for the

dsDNA. MD simulations were performed using the PMEMD module of AMBER14 package[4] and the parm99bsc0. The system was solvated using a 12 Å box of TIP3 waters as well as the counter-ions in the solvent. In each system, a minimization of 10000 steps was performed followed by a heating phase of 200 ps were the temperature was raised from 100 to 300 K. After this thermal equilibration, unrestrained NPT MD simulation was performed for 20 ns in each case.

The number of water molecules surrounding the DNA strands depends on the molecular surface density. At fully hydrated state, the lower the molecular surface density, the larger the number of water molecules per DNA strand in both cases, the ssDNA and the dsDNA. Fig. S7 shows the molecular dynamics simulations for ssDNA and dsDNA.

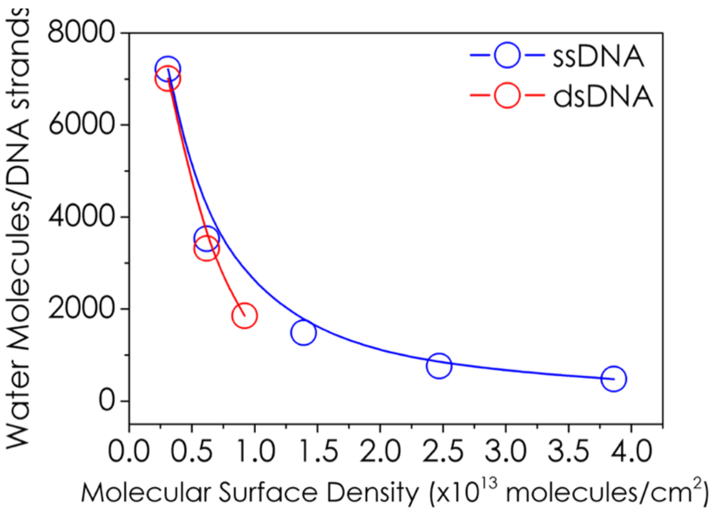

Figure S7 | Molecular dynamics simulations of the number of water molecules per DNA strand. At the fully hydrated state, the water molecules around the DNA strands depends on the molecular surface density: the lower the molecular surface density, the larger the number of water molecules per DNA strand. Blue symbols represent the case of the ssDNA, whereas the red symbols correspond to the dsDNA, the corresponding lines are just a fitting of the calculated values.

In order to account for the hydration dynamics, the hydration/dehydration cycles were simulated by linearly sweeping the number of water molecules from zero to the maximum number per molecule described above. The water uptake by DNA as a function of the relative humidity has extensively been studied in samples consisting of DNA fibers obtained by standard desiccation methods[5]. In these studies, firstly established by Falk and collaborators the water adsorption isotherms closely follow the Brunauer–Emmett–Teller (BET) equation[6]; however, it has been demonstrated that a linear

dependency is a good approximation[5]. Since the calculation of the mass density takes into account the number of water molecules, it was also correspondingly changed.

S6. Molecular Dynamics Simulations. DNA layer thickness

As it was described along the text, the intermolecular forces associated to the crowding effect have a direct impact in the thickness, which increases as the molecular surface density increases. We have used molecular dynamics simulations to calculate the dependency of the thickness with the surface density for both the ssDNA and dsDNA. The Fig. S8 shows the simulated results for the film thickness in nanometers for two different concentrations, $0.31\times 10^{13}\ molecules/cm^2$ and $3.86\times 10^{13}\ molecules/cm^2$ at two different ambient conditions, fully hydrated and high vacuum. The x and y coordinates represent the position in nm for a 16 x 8 nm^2 simulation grid. It is possible to distinguish two main features from these simulations: the mean thickness of the ssDNA layer increases with the surface density and the thickness of the fully hydrated layer is always larger than the corresponding one at high vacuum.

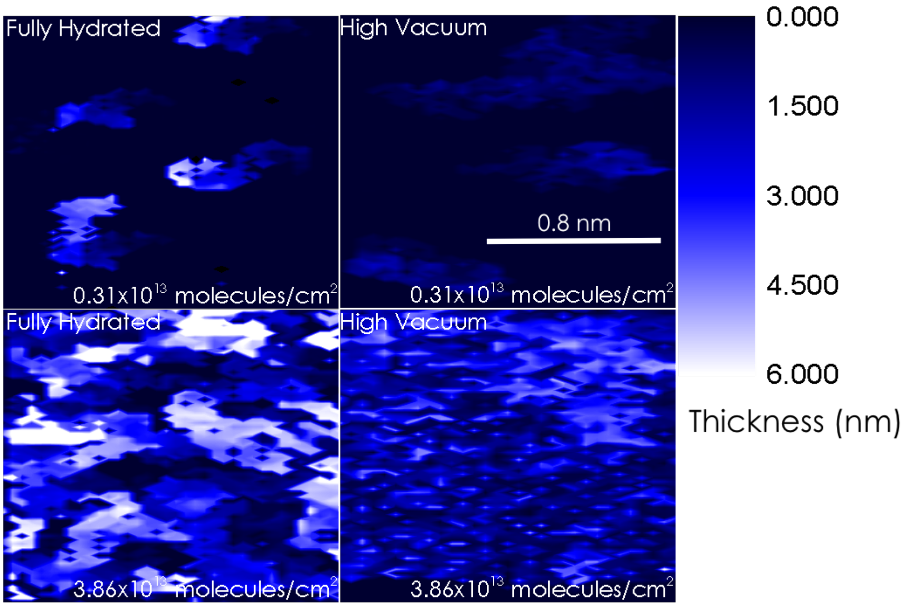

Figure S8 | Molecular dynamics simulations of the ssDNA layer thickness as a function of molecular surface density and ambient relative humidity. At the fully hydrated state, the ssDNA layer is always thicker than the corresponding one at high vacuum. On the other hand, as the surface density increases, the thickness also increases due to the intermolecular forces between the DNA strands.

Fig. S9 shows molecular dynamics simulations of the layer thickness for the dsDNA at two different molecular surface densities: $0.31 \times 10^{13}\ molecules/cm^2$ and $0.62 \times 10^{13}\ molecules/cm^2$ at the same two ambient conditions (fully hydrated layer and high vacuum). By comparing the simulations for both the ssDNA and dsDNA we realize that there are two main differences between them: firstly, the dependency of the dsDNA thickness on the molecular surface density is almost negligible; and the thickness of the dsDNA layer is always larger than the corresponding one of the same grafting density of the ssDNA. The latter can be understood by thinking about the stiffness of the single DNA strand, which is larger in the case of the dsDNA. The persistence length for the dsDNA is known to be larger than the case of the ssDNA. As long as the distance between the molecules decreases, the forces between them increases raising the strands; thus, the dependency on the surface density is related with the collective crowding effect, which is handicapped by the double helix shielding effect due to the force attenuation between the double helices.

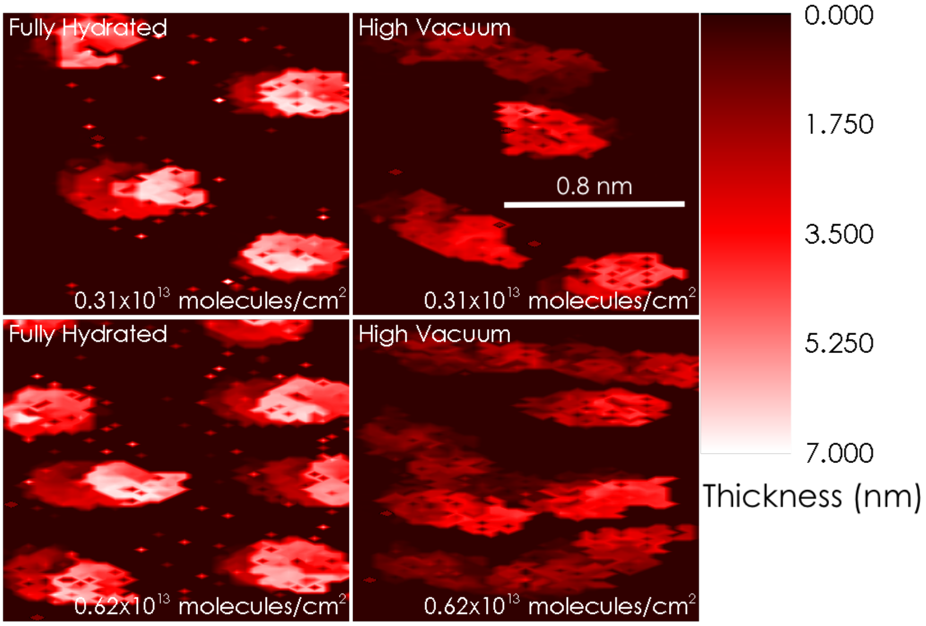

Figure S9 | Molecular dynamics simulations of the dsDNA layer thickness as a function of the molecular surface density and the ambient relative humidity. The dependency of the dsDNA thickness on the molecular surface density is almost negligible due to the double helix shielding effect, whereas the persistence length of the dsDNA is larger than the ssDNA, giving as a result a thicker layer.

In order to study the dynamics of the SAM formation, we simulate the thickness of the layer as a function of the immobilization time for both the ssDNA and dsDNA at fully hydrated state and at high vacuum, Fig. S10. As it was

described above, the thickness of the dsDNA is larger than the ssDNA. From these simulations, it is also possible to see how the dsDNA reaches a saturation value of the thickness in barely two hours, which is indicative of the shielding effect of the double helix. Note also that the thickness of the hydrated layer is larger. This is because of the extra rigidity provided by the water molecules placed around the DNA strands.

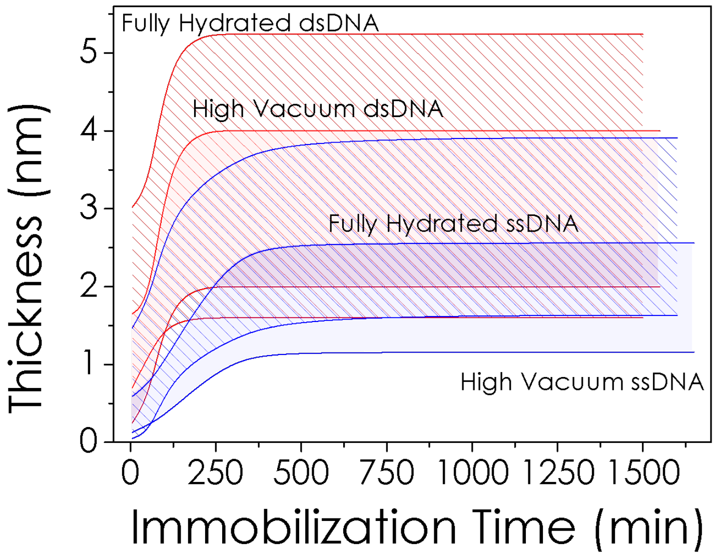

Figure S10 | Molecular dynamics simulations of the DNA layer thickness as a function of the immobilization time. Note that the thickness of the fully hydrated layer (patterned areas red for dsDNA and blue for ssDNA) is larger than the simulated high vacuum conditions. This is attributed to the stiffness supplied by the water molecules placed around the DNA strands. Water acts as a solvent swelling the polyelectrolyte, which should be smaller for the dsDNA.

S7. Finite Elements Simulations. Frequency response of a DNA sensitized Nanomechanical resonator

It is known that the nanomechanical devices are extremely sensitive to changes in both the inertial mass and the stiffness and they have been extensively used as sensors with unprecedented sensitivities. Along the present work we have derived an expression describing a surface density dependent effective Young's modulus of the DNA layer. By using the molecular dynamics simulation method described above we can also calculate the mass density of the fully hydrated layer. Therefore, we have all the necessary elements to simulate the response of a nanomechanical resonator with an attached effective polymer-like layer varying its thickness, Young's modulus and mass

density as function of molecular surface density. Fig. S11 shows the Finite Element Method (FEM) simulations of a cantilever with the same dimensions than the experimental device (500 µm long, 100 µm wide and 1 µm thick). In blue open circles, we can see the effect on the resonance frequency of growing ssDNA layer on top of the cantilever. For low molecular packing the effective Young's moduli are too small to induce a measurable frequency change, shifting the fundamental resonance to lower frequencies only due to the added mass. For sake of major understanding, the shift in the resonance frequency of a pure added mass effect is shown in dashed black line, being always negative. However, as long as the molecular surface density increases both the thickness and the effective Young's modulus of the layer increases, stiffening the cantilever and consequently shifting the resonance to higher frequencies; a pure stiffness effect is shown as dashed green line. The critical molecular surface coverage where the stiffness cancels the negative shifting of the added mass takes place at 30% of surface coverage. The relative frequency shift corresponding to the simulated absorption of the dsDNA layer is also shown in Fig. S11 as red open circles. The relative frequency shift was calculated using the corresponding bare-gold-cantilever frequency as reference. Thus, note that the frequency shift at null dsDNA coverage corresponds to the ssDNA. As it was described in the main text, the effective Young's modulus of the dsDNA is one order of magnitude lower than the ssDNA; therefore, the stiffness effect (dotted green line in Fig. S11) is almost zero, being the added mass contribution (dotted black line in Fig. S11) the main measurable effect for the calculated frequency shift.

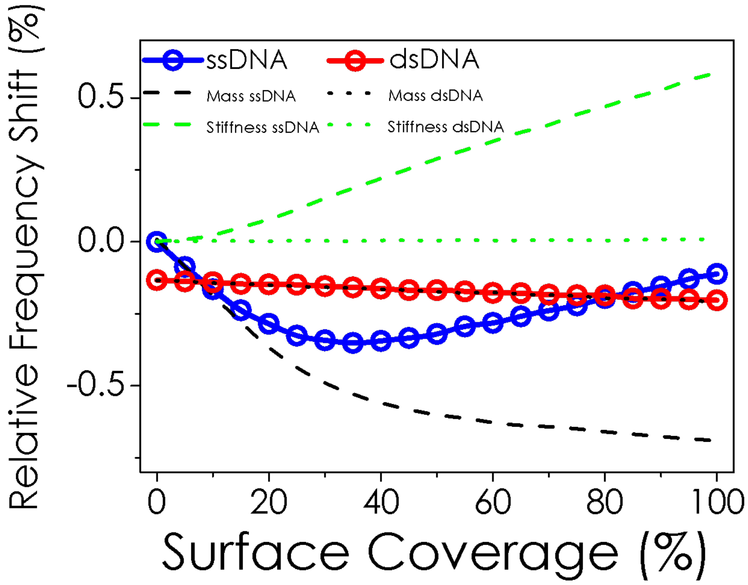

Figure S11 | Frequency response of the functionalized nanomechanical resonator sensor for increasing DNA molecular surface density. Finite elements simulations of a 500 µm long, 100 µm wide and 1 µm thick gold-coated cantilever for increasing ssDNA (blue circles) and dsDNA (red circles) surface coverage form bare gold up to 100%

surface coverage. At low ssDNA molecular surface density, the interaction between the strands is almost negligible, therefore, the resonance is shifted to lower frequencies following the effect of the added mass (black dashed line). However, as long as the molecular surface density increases, the intermolecular forces raise hardening the resonator and shifting the resonance to higher frequencies (green dashed line for pure stiffness effect). The combined effect (blue circles) shows a minimum at a critical surface coverage of about 30% where the increased stiffness due to the intermolecular forces counteracts the added mass. The simulations of the frequency shift of the dsDNA are shown as red circles. The estimated effective Young's modulus for the dsDNA is one order of magnitude lower to the corresponding ssDNA, therefore the stiffness effect is almost negligible (green dotted line), being the mass contribution (black dotted line) the main measurable effect.

S8. Calculation of the Young's modulus of the DNA layer

Simulated bending experiments

Until now we have shown how increasing packing density of DNA molecules has important effects in the layer thickness. As the distance between the strands decreases, there is an increasing attractive collective force due to hydrogen bonding between bases, base-phosphorous interactions, and base stacking. Thus, we expect that the collective elastic properties present a similar dependency. In order to study the elasticity of the DNA layer, we have simulated bending experiments by using a potential mean force using WHAM method[7]. Fig. S12 shows the simulated bending experiments for ssDNA (blue circles series ranging from dark to light blue for increasing molecular surface density) and dsDNA (red circles series ranging from dark to light red). The energy cost increases accordingly with the increasing molecular density packing of the ssDNA, being the corresponding layer of 3.86x10^{13} molecules/cm^2 six times stiffer than the 3.1x10^{12} molecules/cm^2 one; indicating a stiffening effect given by the intermolecular forces. Note that the stiffening of the dsDNA is virtually negligible when compared with the ssDNA.

From the theoretically simulated bending experiments, the effective spring constant[8] of the graphene-DNA system, the inert substrate used in this work is a graphene sheet, k_{eff}^{g-DNA}, is calculated as the second derivative of the energy curves; which will be translated into an effective Young's modulus value, E_{eff}^{g-DNA} by taking into consideration the actual dimensions of the cantilever device used in the experiments, $E_{eff}^{graph-DNA} = {}^{4L^3}/_{wt^3}\, k_{eff}^{graph-DNA}$. Finally, the Young's modulus of the DNA will be decoupled from the system graphene-DNA by solving the following relation:

$$E_{eff}^{g-DNA} = \frac{E_g^2 t_g^4 + E_{DNA}^2 t_{DNA}^4(x) + 2E_g E_{DNA} t_g t_{DNA}(x)\left[2t_g^2 + 2t_{DNA}^2(x) + 3t_g t_{DNA}(x)\right]}{E_g t_g + E_{DNA} t_{DNA}(x)}$$

where the subscripts *g* and *DNA* refer respectively to the graphene and DNA. Note that both the thickness, *t*, and the Young's modulus, *E*, of the DNA layer depend on the molecular surface density, x. This dependency arises from the MD bending data and it has also been experimentally observed in this work.

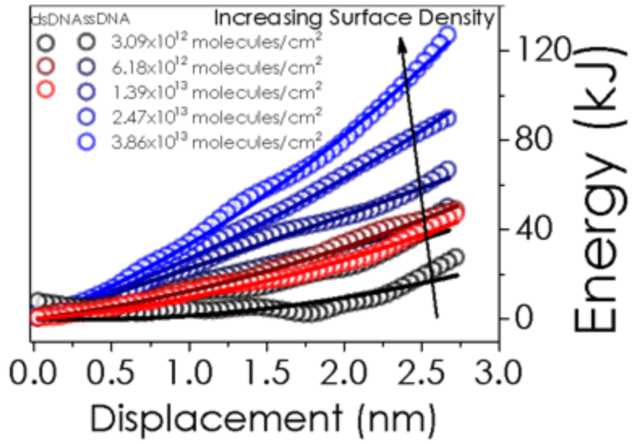

Figure S12 | Simulated bending experiments. Molecular dynamics simulation of the bending experiment for increasing molecular density of ssDNA (from black to blue circles) and dsDNA (from black to red circles). The rising intermolecular force hardens the cantilever increasing the energy needed to bend the structure with the increasing number of molecules. The second derivative of the energy bending curves gives the spring constant of the simulated system, which is used to calculate the effective Young's modulus of the DNA layer. The effective Young's modulus increases with the surface density; however, whereas in the ssDNA layer (blue lines), it reaches a maximum value of about 15 GPa, the effective Young's modulus for dsDNA (red lines) is one order of magnitude lower.

S9. Intermolecular forces

The formation of highly packed single-stranded DNA (ssDNA) monolayers on surfaces and the effect of intermolecular forces among neighboring chains is driven by the intermolecular forces, changing the angle sustained by the DNA strands and the substrate, see the main text. Molecules relate each other via diverse interactions, being a particularly relevant one the mentioned water-mediated hydrogen bonding[9]. A balance of different non-covalent forces governs the structure of this DNA biomolecular layers[10]; in this sense, DNA SAMs could then be understood as a super-structure formed by auto-organized molecules surrounded by water molecules.

Intuitively, the attractive forces stabilize the DNA SAM preserving its structure, these include Watson-Crick hydrogen bonding, base-phosphorous hydrogen bonding, and base stacking (see Fig. S13a-c) which could be understood as the combination of the hydrophobic effect of the bases buried in the interior of the helix exposing the hydrophilic heads (phosphates) to the surrounding water and the Van der Waals base-base interaction. On the contrary, the electrostatic repulsion between the phosphates present along the same strand and from the phosphates corresponding to the near molecules tend to destabilize the structure.

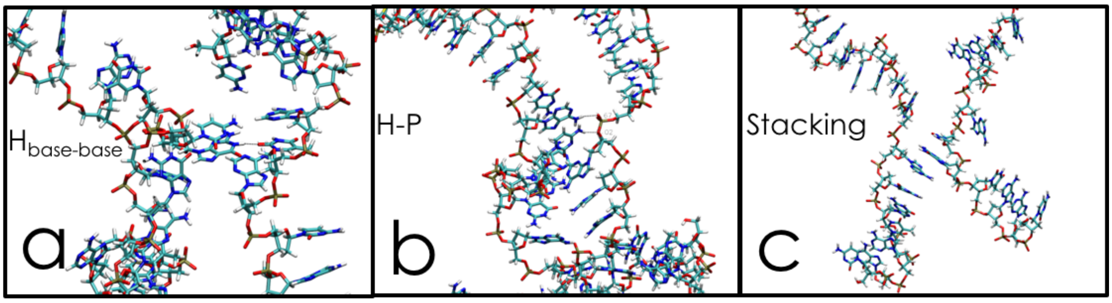

Figure S13 | Intermolecular forces acting in the ssDNA SAM. (**a**), (**b**) and (**c**) Molecular dynamics calculations of the ssDNA attractive interactions. There are three different interactions: Watson-Crick hydrogen bonding, labeled as $H_{base\text{-}base}$; hydrogen phosphate bonding, labeled as H-P; and base stacking

S10. Cantilever Bare-Gold Reference

Fig. S14 shows the hydration/dehydration cycle for different bare-gold cantilevers used as reference in the experiments. As it can be seen from the figure, the repeatability in the resonance frequency shift is very good. Note that there is a small time-drift in these measurements, which is responsible of the non-closing pathway.

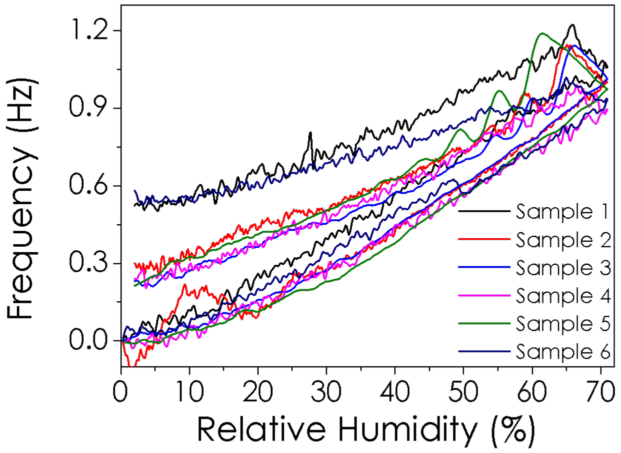

Figure S14 | Cantilever bare-gold reference. Experimental measurement of hydration/dehydration cycle of 6 bare-gold cantilevers. There is a small time-drift in the measurements, responsible of the non-closing pathway. The repeatability is very good.

S11. Thickness Uncertainty

Attending the simulations (commercially available software Schrödinger), the length of a 20-base long ssDNA is 6.4nm, far away from the measured value of 1.2nm, but the measured thickness is still in reasonable agreement with the MD simulations, as can be seen from the following images. The first one corresponds to the length calculation in Schrödinger, and the second one is the fig. S8 of the Supplementary Information.

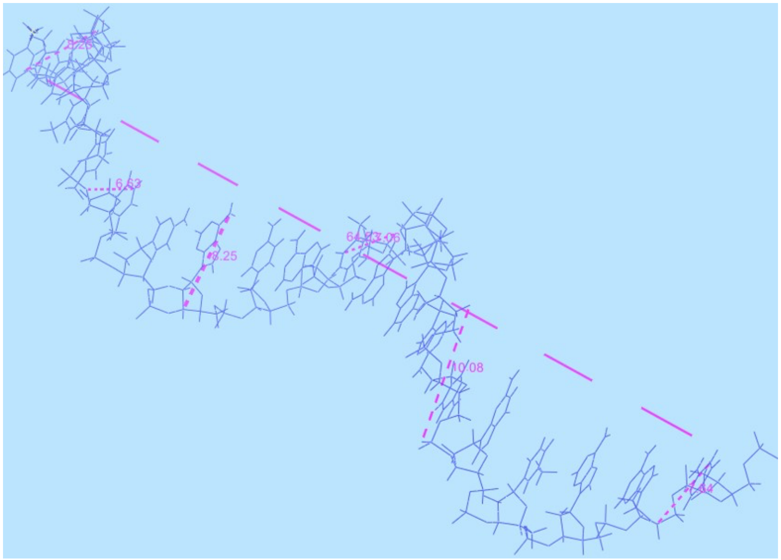

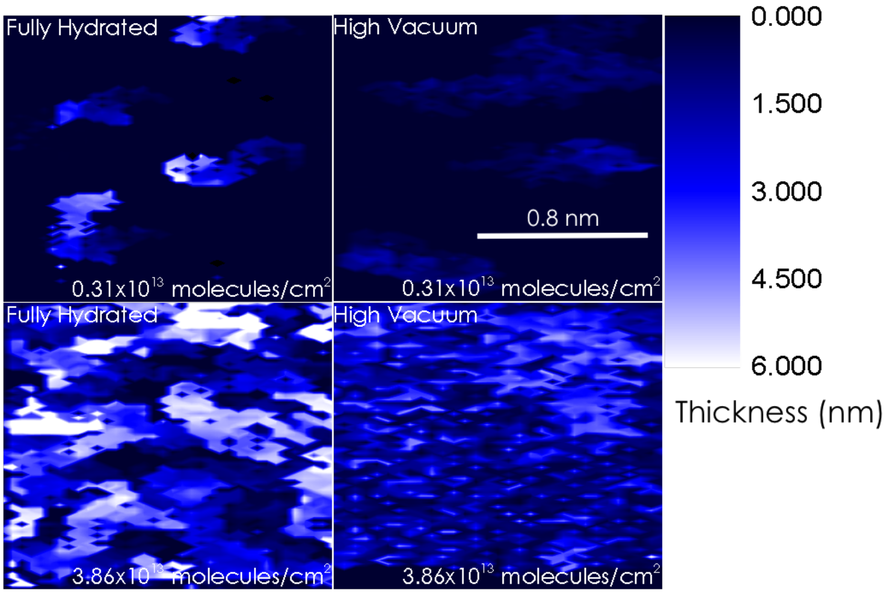

The averaged value of the thickness for MD simulations at $3.86x10^{13}$ molecules/cm^2 is of 2.5 ± 2.1 nm. The dispersion of this value is very large, almost the 100% of the value. Please, note that there is a non-negligible surface area which is not covered by DNA.

At this point, we should take into account the way this thickness was calculated. The final point of the thickness growing curve was measured by AFM at the maximum molecular surface density. We set a DNA step by simply shadowing a gold area and measure the thickness variation. Please, take a look at one of the performed measurements for the grafting density of $6x10^{13}$ molecules/cm^2.

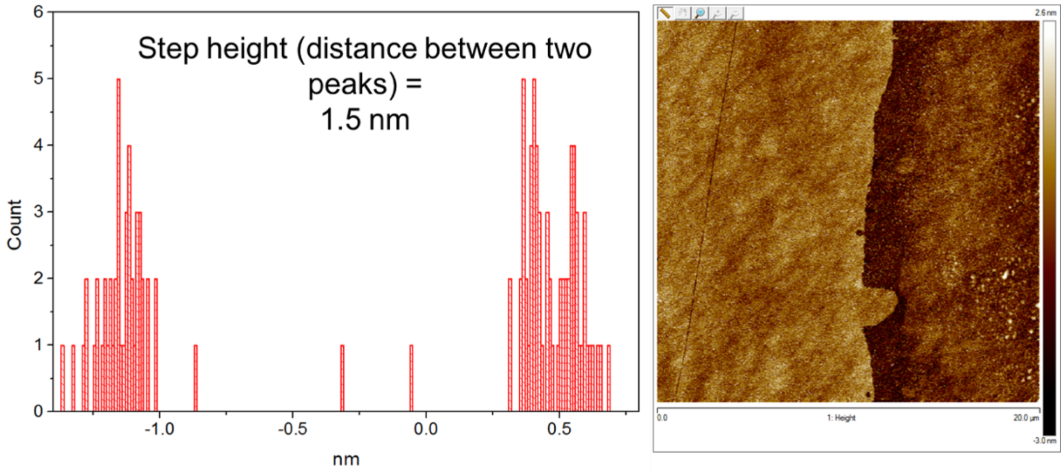

Although all the trends are correct, we attribute the difference among measurements and the MD simulations to the attractive force in between the gold and the bases composing the ssDNA layer, note that the simulations are done on an inert surface (graphene). Nevertheless, it

seems that is too much material to pack into such a small thickness. We really think that the MD simulations could provide a better estimation for the thickness tan the experimental XPS and AFM measurements presented. Note that the AFM measurements averages the thickness measurement in an area equivalent to the tip surface area, and, as it was observed in previous studies, the DNA forms growing coalescent islands. Thus, the AFM averages with the bare surface.

We must take into account the error in the thickness estimation by using AFM. As it was explained above, this error comes from the calibration method employed. The calibration of AFM was made by using a calibration sample trenches of height -21 ± 1 nm. The maximum tolerance after AFM calibration is of 1%. Therefore, the average of our DNA thickness measurements retains and propagates that error, giving as a result 1.2 ± 1.1 nm. This uncertainty propagates to the Young's modulus determination to reach an error of 33% in the value determination.

S12. Error Propagation Calculation

The calibration of AFM was made by using a calibration sample made of trenches of height -21 ± 1 nm. The maximum tolerance after AFM calibration is of 1%. Therefore, the average of our DNA thickness measurements retains and propagates that error, giving as a result 1.2 ± 1 nm.

We can neglect the cantilever's thickness error ($\frac{\Delta t_c}{t_c} \simeq 0.001$), the mass density error as explained in the main text, and the frequency measurement error ($\frac{\Delta f}{f} \simeq 0.05$); therefore, the main error source is coming from the layer thickness estimation. The error propagation in the Young's modulus coming from the thickness error is given by: $\Delta E_{eff}^{DNA} = \sqrt{\left|\frac{\partial E_{eff}^{DNA}}{\partial t}\right| (\Delta t)^2}$; where, as it is explained in the main text, the Young's modulus is calculated as the analytical solution of:

$$\begin{aligned}\Delta\omega/\omega_0 \cong 1/2\left(3\,E_{eff}^{DNA}/E_c - \rho^{DNA}/\rho_c\right)(t^{DNA}/t_c) \\ + 3/8\left[(\rho^{DNA}/\rho_c)^2 + 2\,E_{eff}^{DNA}/E_c\,(4 - \rho^{DNA}/\rho_c) \right. \\ \left. - 7\left(E_{eff}^{DNA}/E_c\right)^2\right](t^{DNA}/t_c)^2\end{aligned}$$

By doing this analysis, assuming a thickness error of 66%, the variation in Young's modulus is of 33%, which is translated to the

calculated values as, for example, $E_{eff}^{DNA}(3.7\times10^{13}\,molecules/cm^2{}_{RH=70\%}) = 12.3 \pm 4.1\ GPa$.

REFERENCES

1 Petrovykh, D. Y., Kimura-Suda, H., Tarlov, M. J. & Whitman, L. J. Quantitative Characterization of DNA Films by X-ray Photoelectron Spectroscopy. *Langmuir* **20**, 429-440, doi:10.1021/la034944o (2004).

2 Koo, K. M., Sina, A. A. I., Carrascosa, L. G., Shiddiky, M. J. A. & Trau, M. DNA-bare gold affinity interactions: mechanism and applications in biosensing. *Analytical Methods* **7**, 7042-7054, doi:10.1039/C5AY01479D (2015).

3 Bustamante, C., Bryant, Z. & Smith, S. B. Ten years of tension: single-molecule DNA mechanics. *Nature* **421**, 423-427 (2003).

4 D.A. Case, J. T. B., R.M. Betz, D.S. Cerutti, T.E. Cheatham, III, T.A. Darden, R.E. Duke, T.J. Giese, H. Gohlke, A.W. Goetz, N. Homeyer, S. Izadi, P. Janowski, J. Kaus, A. Kovalenko, T.S. Lee, S. LeGrand, P. Li, T. Luchko, R. Luo, B. Madej, K.M. Merz, G. Monard, P. Needham, H. Nguyen, H.T. Nguyen, I. Omelyan, A. Onufriev, D.R. Roe, A. Roitberg, R. Salomon-Ferrer, C.L. Simmerling, W. Smith, J. Swails, R.C. Walker, J. Wang, R.M. Wolf, X. Wu, D.M. York and P.A. Kollman *AMBER 2015*. (University of California, 2015).

5 Cagliani, A., Kosaka, P., Tamayo, J. & Davis, Z. J. Monitoring the hydration of DNA self-assembled monolayers using an extensional nanomechanical resonator. *Lab on a Chip* **12**, 2069-2073, doi:10.1039/C2LC40047B (2012).

6 Falk, M., Hartman, K. A. & Lord, R. C. Hydration of Deoxyribonucleic Acid. I. a Gravimetric Study. *Journal of the American Chemical Society* **84**, 3843-3846, doi:10.1021/ja00879a012 (1962).

7 Kumar, S., Rosenberg, J. M., Bouzida, D., Swendsen, R. H. & Kollman, P. A. THE weighted histogram analysis method for free-energy calculations on biomolecules. I. The method. *Journal of Computational Chemistry* **13**, 1011-1021, doi:10.1002/jcc.540130812 (1992).

8 Sader, J. E. a. C., James W. M. and Mulvaney, Paul. Calibration of rectangular atomic force microscope cantilevers. *Review of Scientific Instruments* **70**, 3, doi:DOI:http://dx.doi.org/10.1063/1.1150021 (1999).

9 Boland, T. & Ratner, B. D. Direct measurement of hydrogen bonding in DNA nucleotide bases by atomic force microscopy. *Proceedings of the National Academy of Sciences* **92**, 5297-5301 (1995).

10 Kool, E. T. Hydrogen Bonding, Base Stacking, and Steric Effects in DNA Replication. *Annual Review of Biophysics and Biomolecular Structure* **30**, 1-22, doi:doi:10.1146/annurev.biophys.30.1.1 (2001).
